# Takotsubo Cardiomyopathy in Dextrocardia with Situs Inversus

**DOI:** 10.1155/2020/8844691

**Published:** 2020-08-20

**Authors:** A. Ganes, L. Segan

**Affiliations:** Department of Cardiology, Barwon Health, Bellerine Street, Geelong, VIC 3220, Australia

## Abstract

Takotsubo cardiomyopathy (TTC) is an acute reversible form of left ventricular (LV) systolic dysfunction extending beyond a coronary artery vascular territory usually due to physical or psychological stressors. Dextrocardia with situs inversus is a rare embryologic anomaly whereby the heart and aorta are mirrored on the contralateral side. We describe a case of a 93-year-old female with dextrocardia who presented with chest pain, atrial fibrillation with rapid ventricular response, and transient inferior ST elevation. Coronary angiography demonstrated an eccentric mid right coronary artery (RCA) lesion and apical ballooning consistent with concurrent takotsubo cardiomyopathy (TTC). To our knowledge, this is the first reported case of this dual pathology in a patient with dextrocardia, highlighting the procedural and diagnostic complexity in the setting of a rare anatomicvariant.

## 1. Introduction

Takotsubo cardiomyopathy (TTC) is an acute reversible form of symmetrical left ventricular systolic dysfunction extending beyond a coronary artery vascular territory, often occurring in postmenopausal women and frequently in the context of physical or psychological stressors [1, 2]. It is a recognised acute coronary syndrome (ACS)mimic, accounting for 2 % of ACS presentations [3]. While the aetiology of this condition is incompletely understood, it is believed to be precipitated by an increase in the level of circulating plasma catecholamines [1]. Historically, the diagnosis was made during coronary angiography in the absence of obstructive coronary artery disease (OCAD); however, the coexistence of TTC and OCAD is increasingly recognised [4–7].

We present a rare case of simultaneous TTC and OCAD in a patient with situs inversus dextrocardia, reinforcing that the presence of OCAD should not preclude a diagnosis of TTC. Moreover, the coexistence of TTC and OCAD in a patient with situs inversus dextrocardia raises unique diagnostic and procedural challenges not previously described.

## 2. Case Description

**Figure 1:**
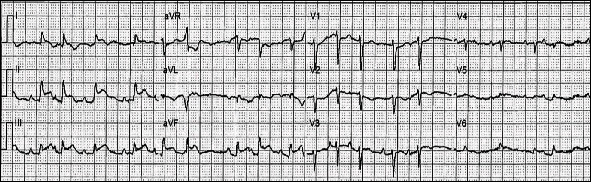
Admission standard 12-lead electrocardiogram (ECG) demonstrating inferior ST elevation, atrial fibrillation with rapid ventricular response, and features associated with dextrocardia (loss of praecordial R wave progression, dominant praecordial S wave).

**Figure 2:**
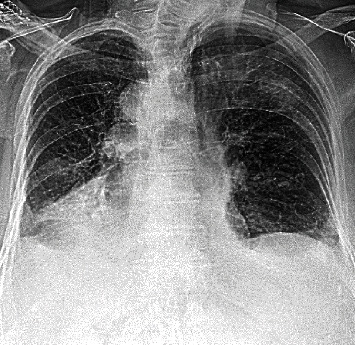
Chest X-ray demonstrating multifocal pneumonia, right-sided aortic arch, cardiac silhouette, and gastric bubble.

**Figure 3:**
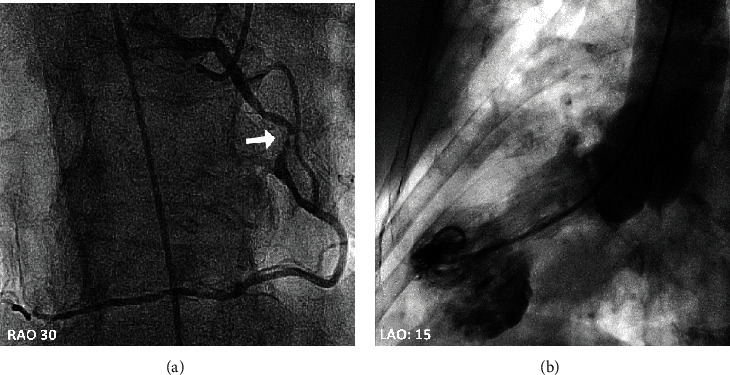
(a) Coronary angiography demonstrating RCA arising from left coronary cusp with an eccentric mid-RCA stenosis (depicted by white arrow). (b) Left ventriculography demonstrating apical ballooning in keeping with TTC.

A 93-year-old female with known situs inversus dextrocardia presented with chest pain, increased work of breathing and a productive cough for the preceding 24 hrs. She was tachypnoeic (respiratory rate of 30 breaths per minute), hypoxic (oxygen saturation of 88% on room air), and in atrial fibrillation (AF) with rapid ventricular response. She was normotensive and afebrile. She had dual heart sounds with no murmurs and coarse crepitations in right upper and lower lung fields. Her admission ECG demonstrated inferior ST-segment elevation (Figure 1) in the setting of AF with rapid ventricular response. Her ECG had hallmark features of dextrocardia (loss of praecordial R wave progression and a dominant S wave). Chest X-ray demonstrated multifocal pneumonia and a rightward-facing cardiac apex, a right-sided aortic arch, and a right-sided gastric bubble consistent with situs inversus dextrocardia (Figure 2). Laboratory investigations revealed an acute inflammatory process with elevated neutrophil count (16.3 × 10^9^*cells*/*L*), C-reactive protein (CRP 293 mg/L) and subsequent sputum culture detecting *Haemophilus Influenzae*. Initial Troponin I was elevated (0.74 *μ*g/L), which incremented to 4.25 *μ*g/L after 6 hours (normal range <0.05 *μ*g/L). Coronary angiography demonstrated an eccentric mid-RCA stenosis (Figure 3(a)) with mild bystander disease. The RCA originated from the left coronary cusp and was engaged with a traditional Judkins Right 4.0 catheter with conventional angiographic image acquisition. The left coronary system (arising from the right coronary cusp) was engaged using a Judkins left 3.5 diagnostic catheter using traditional angiographic angulation. She spontaneously reverted to sinus rhythm at the time of angiography, with resolution of chest discomfort and ECG changes (Supplementary Figure 1). Left ventriculography demonstrated symmetrical apical ballooning with basal sparing consistent with an apical-variant TTC (Figure 3(b)). A decision was made against intervention as the RCA lesion was deemed high risk, the symptoms had resolved with recovery of sinus rhythm and the finding of TTC. Apical modified transthoracic echocardiography (TTE) confirmed severe hypokinesis of the apical segments consistent with left ventriculography. Guideline-directed pharmacotherapy was initiated including an Angiotensin-Converting Enzyme Inhibitor (ACEi) and beta-blocker. Intravenous antibiotics were administered for 48 hours and then de-escalated to oral therapy for multifocal pneumonia with interval improvement in chest X-ray and inflammatory markers (Supplementary Table 1). She was transferred to a subacute rehabilitation unit following a protracted medical admission.

## 3. Discussion

Situs inversus dextrocardia is a rare congenital condition whereby the internal viscera are mirrored to the contralateral side, with an estimated prevalence of <0.0001% in the general population [8, 9]. Undertaking cardiac investigations in the setting of anatomical variations is challenging, requiring careful consideration of impact on diagnostic accuracy and interpretation. Dextrocardia is identified on a standard 12-lead ECG by hallmark features including dominant S waves in praecordial leads with poor R wave progression, right axis deviation, T wave inversion in lead I and a VL and a positive QRS complex in aVR [10]. Right-sided ECG is recommended to aid in interpretation of the praecordial leads [11]. In our case, the right-sided ECG demonstrated appropriate R wave progression in the praecordial leads (Supplementary Figure 1).

ECG findings in TTC and OCAD are often indistinguishable [12] with invasive angiography being the gold standard diagnostic tool [13]. This is further complicated by marked anatomic variation, requiring careful consideration of procedural technique, equipment, and image acquisition to formulate a diagnosis. Literature regarding the approach to invasive coronary angiography insitus inversus dextrocardia is variable, describing a limited user experience with catheter selection, catheter engagement strategy, and modified image acquisition techniques, including mirror image fluoroscopic views and double inversion techniques [14–18]. Acquiring adequate TTE images is also extremely challenging and requires contralateral imaging with a mirror-image technique [19].

Historically, the Mayo Clinic Framework guidelines dictated that a diagnosis of TTC could not be made in the presence of OCAD [20]. Recent literature recognises an increasing incidence of dual pathology in a proportion of presentations, suggesting that these conditions are not mutually exclusive [20–23] and this enhanced understanding is now reflected in contemporary literature [23, 24].

In this case, TTC appeared to be triggered by a combination of pneumonia, tachyarrhythmia, and underlying coronary artery disease. The decision not to intervene on the RCA lesion was made on the basis of multiple physical stressors likely leading to demand ischaemia within a fixed stenosis, with complete symptom and electrographic resolution following the restoration of sinus rhythm. TTC was also likely a consequence of systemic illness and haemodynamic stress.

In cases with coexistent TTC and OCAD, the diagnosis can be clarified with the use of cardiac MRI whereby TTC and myocardial ischaemia have distinctly different characteristics [25].

## 4. Conclusion

We describe a patient with situs inversus dextrocardia presenting with TTC and concurrent OCAD. This case highlights that the presence of coronary artery disease should not preclude a diagnosis of TTC in the appropriate context and the procedural and diagnostic complexities of recognising dual pathology in the setting of situs inversus dextrocardia.

## Supplementary Materials

Supplementary Materials Supplementary Figure 1. (a) Right-sided ECG depicting inferior STE with reciprocal T wave inversion in I, a VL leads. (b) Standard ECG after resolution of atrial fibrillation and inferior ST segment elevation. Supplementary Table 1: trend of biochemistry from admission to discharge. Elevated biochemistry findings are underlined.
