# Supplementary material for: Takotsubo Cardiomyopathy in Dextrocardia with Situs Inversus
Source: Case Rep Cardiol. 2020 Aug 20;2020:8844691. doi: 10.1155/2020/8844691 (PMC7455845; doi:10.1155/2020/8844691)
Supplement: Supplementary Materials — Supplementary Figure 1. (a) Right-sided ECG depicting inferior STE with reciprocal T wave inversion in I, a VL leads. (b) Standard ECG after resolution of atrial fibrillation and inferior ST segment elevation. Supplementary Table 1: trend of biochemistry from admission to discharge. Elevated biochemistry findings are underlined. [file 8844691.f1.docx]

Supplementary Material

**A**


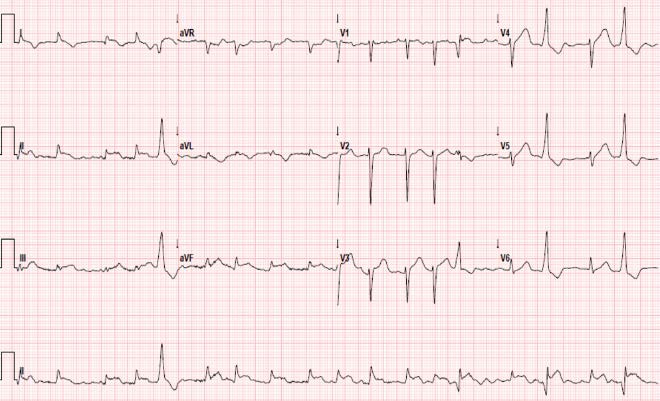


**B**


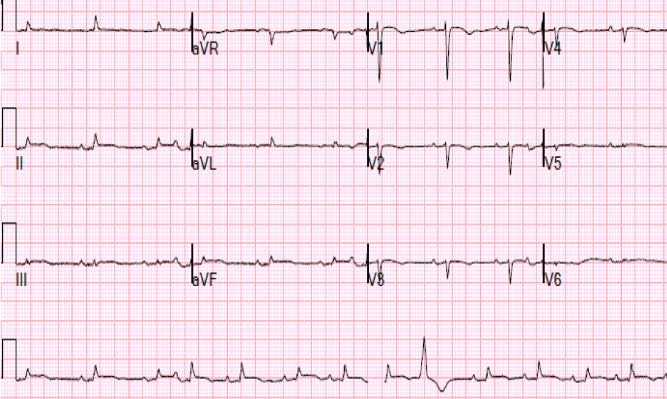

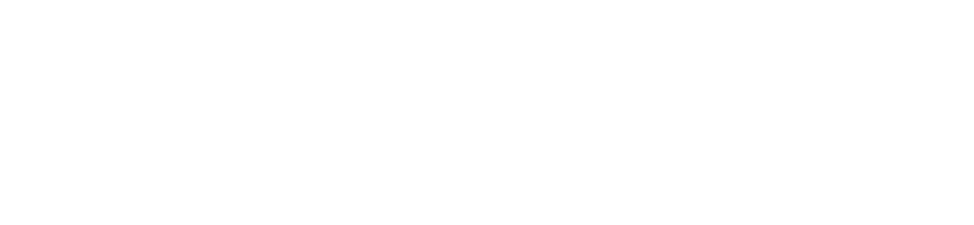


Supplementary Figure 1: (A) Right sided ECG depicting inferior STE with reciprocal T wave inversion in I, aVL leads. (B) Standard ECG after resolution of atrial fibrillation and inferior ST segment elevation

|  | 25/04/2019 (10:30) | 25/04/2019 (16:30) | 26/04/2019 | 30/04/2019 | 04/05/2019 |
| --- | --- | --- | --- | --- | --- |
| Neutrophil Count (x 109 cells/L) | 16.3 |  | 12.4 | 11.9 | 7.5 |
| C-Reactive Protein (mg/L) | 293 |  | 239 | 103 | 15.9 |
| Troponin I (µg/L) | 0.74 | 4.25 |  |  |  |

Supplementary Table 1: Trend of biochemistry from admission to discharge. Elevated biochemistry findings are underlined.
